# Associations between Children’s Physical Activity and Neighborhood Environments Using GIS: A Secondary Analysis from a Systematic Scoping Review

**DOI:** 10.3390/ijerph19031033

**Published:** 2022-01-18

**Authors:** Melody Smith, Suzanne Mavoa, Erika Ikeda, Kamyar Hasanzadeh, Jinfeng Zhao, Tiina E. Rinne, Niamh Donnellan, Marketta Kyttä, Jianqiang Cui

**Affiliations:** 1 School of Nursing, The University of Auckland, Auckland 1142, New Zealand; jinfeng.zhao@auckland.ac.nz (J.Z.); n.donnellan@auckland.ac.nz (N.D.); 2 Melbourne School of Population and Global Health, University of Melbourne, Melbourne, VIC 3053, Australia; suzanne.mavoa@unimelb.edu.au; 3 MRC Epidemiology Unit, University of Cambridge, Cambridge CB2 0SL, UK; erika.ikeda@mrc-epid.cam.ac.uk; 4 Department of Built Environment, Aalto University, 00076 Aalto, Finland; Kamyar.hasanzadeh@aalto.fi (K.H.); tiina.e.rinne@aalto.fi (T.E.R.); Marketta.kytta@aalto.fi (M.K.); 5 School of Engineering and Built Environment, Griffith University, Brisbane, QLD 4222, Australia; jj.cui@griffith.edu.au

**Keywords:** geographic information systems, youth, active travel, walking, cycling, play, MVPA, health geography, adolescent, children’s geographies

## Abstract

Regular participation in physical activity is essential for children’s physical, mental, and cognitive health. Neighborhood environments may be especially important for children who are more likely to spend time in the environment proximal to home. This article provides an update of evidence for associations between children’s physical activity behaviors and objectively assessed environmental characteristics derived using geographical information system (GIS)-based approaches. A systematic scoping review yielded 36 relevant articles of varying study quality. Most studies were conducted in the USA. Findings highlight the need for neighborhoods that are well connected, have higher population densities, and have a variety of destinations in the proximal neighborhood to support children’s physical activity behaviors. A shorter distance to school and safe traffic environments were significant factors in supporting children’s active travel behaviors. Areas for improvement in the field include the consideration of neighborhood self-selection bias, including more diverse population groups, ground-truthing GIS databases, utilising data-driven approaches to derive environmental indices, and improving the temporal alignment of GIS datasets with behavioral outcomes.

## 1. Introduction

Regular participation in physical activity is essential for children’s physical, mental, and cognitive health [1,2]. Strong evidence exists for the link between accumulating an average of 60 min of moderate-to-vigorous physical activity (MVPA) daily with improved health [1]. Children can accumulate MVPA in a variety of ways, including through organized sports, unstructured play, and walking or wheeling to and from places (active travel) [3]. Conversely, an increased time spent sedentary (e.g., recreational screen time, television viewing, car travel) is negatively associated with child health [1]. While some exceptions exist, physical activity, play, and active travel in children are generally low internationally [4,5].

In the last two decades, considerable work has been undertaken to understand factors associated with physical activity using a socio-ecological lens. The early work of Sallis and colleagues [6,7] was especially useful to contextualize how varying social and environmental features might impact physical activity and to highlight areas for improvement. In particular, the role of neighborhood design and “walkability” (e.g., higher levels of street connectivity, mixed land use, retail floor area ratio, and population density in a given area) received increasing focus [8,9]. A now well-established body of research clearly demonstrates associations [10,11,12] and causal relationships [13] between neighborhood features and residents’ physical activity, including for children.

Neighborhood environments may be especially important for children who are more likely than adults to spend time in the environment proximal to home [14,15]. Despite a heterogeneous evidence base, consistent findings have been observed with regard to the importance of walkability (especially street connectivity, population density, and diversity in land use) [16,17], infrastructure for walking and wheeling [18], and the availability and accessibility of destinations to be active (e.g., parks, playgrounds, natural spaces, schools) [16,19] for supporting physical activity. Numerous co-benefits exist when environments are designed to enable children’s physical activity, including supporting planetary health (e.g., through reducing air pollution via shifting from motorized to active travel modes [20,21,22]). Indeed, encouraging active travel has been suggested as a “planetary health intervention” recognizing the multiple pathways through which human travel behaviors and planetary health are linked [23]. Children’s physical activity tracks over the lifespan [24,25], so establishing healthy physical activity habits, including active travel, early in life can have a long-standing impact on both human and planetary health.

Alongside this growing evidence base, an increased sophistication and complexity in the measurement of neighborhood environments has occurred. There is a recognition that the approach used to characterize environments can impact the knowledge generated. For example resident perceptions of environmental features may differ considerably from objective assessments of those features [26]. Consequently, there exists a risk of “masking” relationships, where the body of evidence does not take differing measurement approaches into account. Care must be taken to consider how evidence might differ across research using different environmental measurement approaches.

Geographic information system (GIS) approaches to quantifying and evaluating features within neighborhood and health research have burgeoned. A key strength of GIS in this context is the ability to generate consistent measures, enabling comparability across geographies and population groups. Our recent review explored how GIS had been used to define and describe neighborhood environments in research exploring children’s physical activity and related outcomes [27]. A considerable diversity in both measurement approaches and the reporting of methods was identified; recommendations from the review included the need for greater geographic diversity in the evidence, and an improved consistency and transparency in the reporting, aligning with earlier calls for improving the evidence base [28]. As this previous review was focused on measurement, there was not the opportunity to discuss or reflect on the findings of the studies included. The aim of this short communication is to describe the associations observed in the literature sourced. In doing so, we provide an update to the extant evidence base [10,12,16,29] with a specific focus on the GIS measurement of children’s environments.

## 2. Methods

The full review protocol was registered on the Open Science Framework on 28 October 2019 (https://osf.io/7wgur/ (accessed on 7 January 2022)) and is also detailed elsewhere [27]. A brief overview is presented here following the Preferred Reporting Items for Systematic reviews and Meta-Analyses extension for Scoping Reviews (PRISMA-ScR) Checklist criteria [30].

### 2.1. Information Sources, Search Terms, and Search Strategy

GEOBASE, Scopus, PubMed (includes MEDLINE), and Social Sciences Citation Index were searched using terms under three categories: Method (e.g., GIS), Population (e.g., child), and Outcome (e.g., built environment). The following is an example of the full electronic search strategy for PubMed: (GIS OR “geographic information system*” OR model*ing OR geospatial OR spatial) AND (child OR child’s OR children OR children’s OR “elementary school*” OR “primary school*” OR “intermediate school*” OR “junior school*” OR “middle school*” OR youth OR “young people”) AND (“activity space*” OR neigh * AND hood OR “built environment” OR “natural environment” OR “home range” OR “home zone” OR territory OR “living environment” OR “residential environment” OR “action space” OR “geographical context” OR “exposure area” OR “urban environment”) AND ((“2006/01/01”[PDat]: “2019/10/29”[PDat]) AND Humans[Mesh] AND English[lang]).

### 2.2. Eligibility Criteria

Studies were eligible at the searching stage if they were: (1) peer reviewed articles published in academic journals, (2) published in the English language, (3) conducted with human populations, and (4) published between 1 January 2006 (to align with the emergence of literature using GIS for delineating neighborhoods) and 15 November 2019.

### 2.3. Selection of Sources of Evidence

After removing duplicate articles, titles and abstracts of all articles retrieved were screened for inclusion. Studies were eligible for inclusion at the screening stage if they used GIS to measure neighborhood environments and included children (defined as aged 5–13 years). Studies were excluded if they: (1) did not include a GIS-based measure of the neighborhood environment, (2) did not include children, or (3) used area-level measures greater than the neighborhood scale (e.g., towns, cities, regions). Duplicate screening was conducted for a random 10% selection of all articles identified at the search stage. Full text articles were then sourced for all “eligible” articles and for those where it was not clear whether they met the inclusion criteria. At the full-text stage, articles were included if they met the criteria above, and additionally: (1) described the methods used to generate the GIS-based measure of neighborhood environments, (2) included a physical activity outcome measure, or focused on the PA-environment relationship, and (3) provided descriptive information about the GIS-based neighborhood environment outcome (in graphical, narrative, or tabular format). Of note, only articles that included a physical activity outcome were included, and those with related measures only (e.g., body mass index) were excluded.

### 2.4. Quality Assessment

The Mixed Methods Appraisal Tool (MMAT) [31,32,33] was used to assess study quality due to its flexibility in assessing varying research designs (e.g., quantitative non-randomized, quantitative descriptive, mixed methods). Evaluation criteria and summary scores were calculated following the MMAT protocol. Quality assessment was duplicated for a random 10% subset of articles.

### 2.5. Data Charting and Synthesis

Descriptive data of studies included were extracted in duplicate. For the purpose of this examination, key study characteristics, physical activity measurement, and study findings relative to GIS-measured environmental variables were extracted and a narrative description of findings was generated. This study focused on built environments, and thus data on characteristics of the social environment were not extracted unless they were directly related to the GIS findings.

## 3. Results

**Figure 1:**
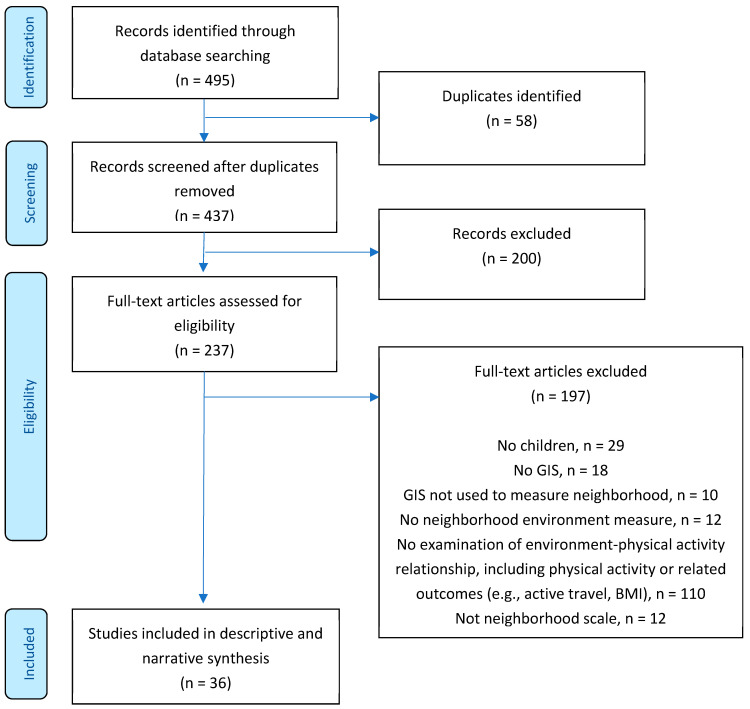
Preferred Reporting Items for Systematic Reviews and Meta-Analyses (PRISMA) flow diagram for articles identified, screened, and included in the review. Note: BMI = body mass index, GIS = geographic information systems.

Figure 1 shows the flow chart for the studies included and excluded at each stage of the review process. Table 1 shows the descriptive information for all studies included, key findings, and MMAT scores. The study quality varied, with MMAT scores ranging from 2 to 5 (possible range 1–5). Article quality scores were most commonly reduced due to a lack of clarity or information on study methods and population representativeness.

Overall, findings showed evidence to support relationships between activity behaviors and street connectivity [36,41,42,47,51,56,58,66], with differential relationships observed by age and sex [38,44], and one study showing a negative relationship in females only [34]. Similarly, generally consistent positive relationships between activity and residential density were found [15,41,42,47,50,52,61,66], with the exception of two studies [15,37], one of which examined the proportion of child population in relation to active school travel [15]. Diversity in land use was positively related to physical activity [34,66], and both the density of entertainment facilities [41] and public transit (school-aged girls only) [38] were all positively related with activity behaviors. Walkability was positively associated with activity behaviors in five studies [41,48,55,64,68] (one found a significant positive relationship in low SES areas only [48]), and physical inactivity was associated with walkability in one study [56].

Inconsistent findings were observed for physical activity facilities (including parks, playgrounds, and outdoor spaces). Positive results were found between activity behaviors and physical activity facilities [36,61,65,68], including pay facilities (males only) [35], public open spaces (school-aged females and preschool children only) [38], parks [52,61,66,68], green space [57], and sports fields [59]. However, a number of negative relationships were observed, including for physical activity facilities/sites [45,66] and a higher proportion/area of parks or green space [15,50,56]. Similarly, food outlet density was both positively [50] and negatively [57] associated with activity behaviors.

The density of main streets (exemplifying less safe traffic environments) was negatively related to activity behaviors in one study [37], and another showed positive relationships for traffic safety infrastructure, with differential findings observed for the time of day and population group (i.e., traffic/pedestrian lights were significant in younger girls only, slow points significant for younger boys before school, and speed humps significant in adolescent boys after school [44]).

Walking or cycling track length (including multi-use path space) was associated with activity behaviors (particularly walking or cycling) in four studies [44,45,51,59], while a fifth found a negative association between cycling infrastructure and children’s license for independent mobility [69]. Distance to school was negatively related to active school travel [42,53,62,66].

## 4. Discussion

The aim of this short communication was to describe the associations observed between GIS-derived environmental features and children’s activity behaviors, drawing from a systematic scope of the literature. In doing so, we have provided an updated review of the extant evidence [10,12,16,29], with a targeted focus on the GIS measurement of children’s environments. Findings align with previous systematic reviews examining environmental associates of children’s physical activity behaviors [10,12,16,29]. While some inconsistencies exist, together this body of literature supports the need for neighborhoods that are well connected, have higher population densities, and have a variety of destinations in the proximal neighborhood to support a range of physical activity behaviors in children. In line with previous reviews [11,70,71], a shorter distance to school and safe traffic environments were significant factors in supporting children’s active travel behaviors. These features are interconnected and speak to the importance of comprehensive urban design approaches that embrace concepts such as walkable neighborhoods [72], livable neighborhoods [73], 15 min cities [74], and 20 min neighborhoods [75]. Across these concepts, having a range of destinations of importance within walkable distances from residential homes is fundamental. Having a sufficient population density to warrant the required transport infrastructure and destination diversity, and to support social cohesion and connection, is also intrinsic to these concepts, meaning that inequities may exist by urbanicity. Increasing attention is also focusing on the importance of low-traffic neighborhoods for increasing physical activity (especially active travel) through increasing safety from traffic and by improving social connection and cohesion [76,77,78]. While this review did not focus on social aspects of supporting children’s PA, social cohesion and connection have been previously identified as important for facilitating children’s physical activity [11,79], so are important co-benefits of these approaches. Ultimately, findings from the current review add evidence to the growing evidence base, demonstrating that connected and comprehensive approaches to urban design are needed to encourage and support children’s physical activity.

Considerable potential also exists for improving planetary health through these environmental approaches [21,22,23]. The transport sector plays a significant role in greenhouse gas emissions [22], and urban design that prioritizes motorized transport can contribute to the urban heat island effect [80]. Shifting the prioritisation of land use away from roads towards connecting communities and providing infrastructure that facilitates active travel is likely to make a meaningful contribution to improving planetary health and achieving the sustainable development goals [81]. Supporting a generation of young children to develop physical activity habits (including active travel) early in life is likely to have a sustained impact on their health and that of their planet [24,25].

A considerable heterogeneity in GIS methods was observed across the studies in this review, and substantial variability in the reporting of GIS methods was found. The GIS database availability and quality limited the body of evidence, with studies purchasing commercial (and potentially incomplete) databases, triangulating a range of data sources to generate measures, and using temporally mismatched datasets to the outcome being measured. Only one study noted “ground truthing” through phone calls and physical visits to food outlet locations [82]. Such ground truthing is particularly important for retail and food outlets, where a higher turnover may occur than changes to the physical activity infrastructure. It is unclear whether ground truthing is as important for physical activity facilities and destinations as it is for food and retail settings. On-the-ground checks for a full GIS dataset are not realistic or feasible (and would negate the need for estimated measures). A more feasible approach is to generate a random subset of the full GIS dataset for ground truthing. However, no recommendations for an optimal proportion for resampling exist. Precedents in child health and environment literature include Huang, Brien [83] physically confirming a random 10% selection of bus stops in a study using Google Street View to measure outdoor advertising around schools, and Vandevijvere, Sushil [84] randomly selecting 1% of 8403 geocoded food outlets and confirming details via telephone; however, the authors are unaware of this occurring with specific regard to physical activity destinations.

Walkability (determined from street connectivity, residential density, land use mix, and sometimes the retail floor area ratio) [85] was the most common index in the literature identified. Other indices were used with varying degrees of rationale for determining and calculating the index. For example, DeWeese et al. [50] used latent class profiling to generate clusters of environmental features associated with BMI, and Ikeda et al. [53] developed a latent variable “active mobility environment”, based on correlations of environmental features in structural equation modeling. Future research in this area is warranted, particularly for different physical activity outcomes, and in different socio-demographic groups.

The reporting of physical activity measurement methods was considerably better than the reporting of GIS measurement in the literature sourced. This may reflect the established state of the field of physical activity research, compared with the relatively recent emergence of research using GIS-derived measures to understand associations between environments and physical activity. Even so, considerable differences in physical activity measurements were observed across studies, further limiting a clear understanding of associations between children’s physical activity and their environments. An increased conceptual matching of the physical activity behavior (e.g., active travel) and environmental features assessed (e.g., active travel infrastructure) is needed to increase the specificity and sensitivity in understanding PA–environment links [86].

A number of other study design strengths and limitations were identified. Numerous studies had representation from ethnically and socio-economically diverse population groups [34,35,36,39,40,41,42,43,44,46,50,53,56,57,60,62,63,64,66,67,68], and some had large and/or representative samples [34,35,36,37,43,47,55,56,58,61,63]; however, evidence was predominantly from the USA [34,35,36,39,40,41,42,43,46,47,50,63,64,68] and there was no literature related to disabled children. Heterogeneity in study environments was encouraged through stratified neighborhood/area sampling in a number of studies. Two studies reported excluding child participants who had recently moved to the neighborhood [54,87], improving the sensitivity and reducing the impact of reactivity on the shifting of physical activity behaviors. Neighborhood self-selection was rarely noted, albeit this may be less important for children than adults, who have more control over where they live. The consideration of children living in more than one home was not noted. A lack of consideration of clustering (e.g., at school level) was a limitation of the body of literature.

## 5. Conclusions

Despite a heterogeneous evidence base, consistent findings were observed that add weight to existing evidence for the essential role of neighborhood environments in promoting children’s physical activity behaviors. Findings highlight the need for neighborhoods that are well connected, have higher population densities, and have a variety of destinations in the proximal neighborhood to support children’s physical activity behaviors. A shorter distance to school and safe traffic environments were significant factors in supporting children’s active travel behaviors. Areas for improvement in the field include the consideration of neighborhood self-selection bias, including more diverse population groups, ground-truthing GIS databases, utilising data-driven approaches to derive environmental indices, and improving temporal and conceptual alignment of GIS datasets with behavioral outcomes.

## Figures and Tables

**Table 1:** Study characteristics, key findings, and quality assessment scores.

Author (Year); Country	No. of Participants; Sex (% Female); Age in Years (y)	Socio-Economic and Ethnicity Characteristics	Physical Activity Outcome(s)	Key Findings *	MMAT Score
Boone-Heinonen and Gordon-Larsen [34]; USA	12,701 at both time points; 51%; 11–22 y in wave 1 (1994/1995), 18–26 y in wave 3 (2001/2002)	Parental household income at wave 1 (mean ± SD): USD43,100 ± 1500; Ethnicity: White 68%, Black 16%, Asian 4%, Hispanic 12%; Highest parental education: Some college or higher 54%	MVPA (self-reported weekly frequency of skating, cycling, exercise, and active sports at wave 1, modified at wave 3 to include age-appropriate activities)	MVPA was positively related to landscape diversity in all participants, and negatively related to street connectivity in females only.	4
Boone-Heinonen, Guilkey [35]; USA	12,701 at both time points; 51%; 11–22 y in wave 1 (1994/1995) 18–26 y in wave 3 (2001/2002)	Parental household income at wave 1 (mean ± SD): USD43,100 ± 1500 ; Ethnicity: White 68%, Black 16%, Asian 4%, Hispanic 12%; Highest parental education: <High school 15%, High school/GED 31%, Some college 29%, College or greater 25%	MVPA (self-reported weekly frequency of skating, cycling, exercise, and active sports at wave 1, modified slightly at wave 3 to include age-appropriate activities)	MVPA was higher with greater PA pay facilities in males only.	5
Boone-Heinonen, Popkin [36]; USA	17,659; 50%; 11–22 y	Significant differences in income tertile by urbanicity, higher proportion of tertile 1 and lower proportion of tertile 3 in high-urban compared with lower-urbanized areas (direction of tertiles was unclear); Urbanicity: non-urban 39%, low-urban 36%, high-urban 24%. Significant differences in education level by urbanicity, lower education levels in high-urban compared with lower-urbanized areas	MVPA (self-reported weekly frequency of skating, cycling, exercise, and active sports)	MVPA was associated with intersection density in 1 km buffer (all in non-urban areas, males in high-urban areas), and count of PA resources in 3 km buffer (all in low-urban areas). Associations with weighted counts were similar to counts within 1–5 km.	5
Bringolf-Isler, Grize [37]; Switzerland	1081; 49% (children), 54% (adolescents); 6–7 y, 9–10 y, 13–14 y	Maternal education: low 16%, medium 48%, high 36%; Car ownership: none 20%, one 66%, two or more 16%	Vigorous outdoor play (parent-reported, daily average)	Vigorous outdoor play was negatively associated with main street density, population, and building density in the 100 m home buffer. Comparable results were observed using 100 m, 200 m, and 500 m buffers.	2
Buck, Kneib [38]; Germany	400; 52%; 2–9 y	Children living in urban environments only	MVPA (min/day, accelerometer)	MVPA was associated with the availability of public open spaces (school girls, pre-school children), public transit (school girls), and higher street connectivity (school girls). Stable results were found within a network-distance using kernel intensity measures from 750 m up to 1.5 km for school children and from 500 m up to 1 km for pre-school children. Different results were observed by buffer size using the simple intensity approach.	4
Cain, Millstein [39]; USA	955; 50%; 6–16 y	Based on the recruitment strategy, approximately half of the participants should be from low income neighborhoods, and half from high income neighborhoods; Ethnicity: non-White 31% (children) and 33% (adolescents); Parent education: college degree 68% (children) and 64% (adolescents)	Walking and cycling to specified locations (parent-reported for children, self-reported for adolescents; average of usual frequency of trips) Average daily minutes of MVPA (accelerometer, non-school hours) Average daily minutes of MVPA in the neighborhood (children only, linking parent-completed daily location logs to accelerometer data, non-school hours) Neighborhood PA (parent-reported for children, self-reported for adolescents)	After adjusting for GIS-defined walkability, numerous observed microscale environmental variables were related to active travel and neighborhood PA.	3
Carlson, Mitchell [40]; USA	528; 50%; 12–16 y	50% resided in high-income neighborhoods; Ethnicity: White non-Hispanic 70%; Home neighborhood walkability: high-walkability 46%	Time and MVPA in specified locations (GPS and accelerometer)	MVPA in one location was mainly independent of MVPA in other locations (i.e., no compensation effect), except for higher at-school MVPA (less at home and other location MVPA) and higher home neighborhood MPVA (more at home MVPA).	4
Carlson, Saelens [41]; USA	690; 51%;12–16 y	Based on the recruitment strategy, approximately half of the participants should be from low income neighborhoods, and half from high income neighborhoods; Ethnicity: White non-Hispanic 69%; Highest parental education: college degree 64%; Marital status: Parent married or living with partner 84.0%; Household car ownership (mean ± SD): 2.5 ± 1.0	Walking and cycling travel time (min/day, GPS and accelerometer) Active travel mode share (daily walking + cycling minutes/total daily travel time × 100, GPS and accelerometer)	Walking time and active travel mode share were associated with residential density, intersection density, entertainment density, and walkability. Cycling time was associated with intersection density and walkability.	3
Carlson, Sallis [42]; USA	294; 47%; 12–16 y	Based on the recruitment strategy, approximately half of the participants should be from low income neighborhoods, and half from high income neighborhoods; Ethnicity: Non-Hispanic Caucasian 69%; Highest parental education: college degree 62%; Marital status: married or living with partner 85%; Parental employment status: full-time 53%; Vehicles/driver in household (mean ± SD): 1.07 ± 0.39	Active travel to/from school on average school week (self-reported), classified as none, occasional (1–4 trips), and habitual (5–10 trips)	Active travel to/from school was positively associated with street connectivity around home, residential density around home, and residential density around school; and negatively associated with distance to school. The odds of travelling actively occasionally or habitually reduced to 0.60 and 0.24, respectively, for every additional km in distance to school.	4
Carroll-Scott, Gilstad-Hayden [43]; USA	1048; 53%; Mean ± SD: 10.9 ± 0.8 y	Free/reduced lunch eligibility 77%; Not food-secure 11%; Ethnicity: Non-white 89%; Primary language at home: not English 38%	Frequency of exercise (self-reported, PACE PA item) Number of hours of weekday screen time (usual duration of TV, video game, and computer (for fun) time on weekdays)	Screen time was negatively associated with living in more affluent neighborhoods.	4
Carver, Timperio [44]; Australia	446 at both time points; 49% (children), 57% (adolescents); 8–9 y and 13–15 y in 2004; 10–11 y and 15–17 y in 2006	Primary language at home: not English 38%	Frequency of walking/cycling trips per week, change in walking/cycling trips per week over time (parent-reported for children, self-reported for adolescents) Mean minutes per day of MVPA and change over time (accelerometer)	Change in active travel was associated with the number of traffic/pedestrian lights (in younger girls), length of walking tracks (younger and adolescent girls), and intersection density (adolescent boys). Change in MVPA was associated with slow points (younger boys before school) and speed humps (adolescent boys after school).	3
Carver, Timperio [45]; Australia	640 (411 primary school-aged, 229 secondary school-aged); 51%; Mean ± SD: 11.6 ± 2.0 y	Primary-school-aged: urban 72% urban; Secondary-school-aged: urban 50%	Cycling at least once per week (self-reported)	The odds of cycling at least once per week were negatively associated with the number of sports facilities within the 5 km buffer and positively associated with living in neighborhoods with the top tertile of length of bike paths in 5 km buffer.	3
Coughenour and Burns [46]; USA	71 (26 children aged 6–18 y); 66%; 6–94 y	Annual household income (N = 42 out of 44, 95%): <USD75,000; Ethnicity: Hispanic 39%; Black 27%; Caucasian 25%	MVPA (parent-reported moderate vigorous PA, such as “pushing a vacuum or climbing 1 flight of stairs” and “running, lifting heavy objects, and strenuous sports”)	No significant difference was observed in those meeting weekly PA recommendations by opportunities for PA in the neighborhood.	3
Dalton, Longacre [47]; USA	1552; 52%; 12–17 y	Annual household income: >USD$75,000 38%; Ethnicity: Non-Hispanic Caucasian 92%; Highest parental education: bachelor degree or higher 38%; Single-parent household 19%	Active travel to/from school, defined as walking or biking to or from school at least 1 day per week during one or more season (self-reported)	Active school travel was associated with higher residential and intersection densities and lower distance to school (81% who lived within 1 mile of school were active travelers versus 30% of those who lived 2–3 miles from school).	4
De Meester, Van Dyck [48]; Belgium	637; 50%; 13–15 y	Education level: college degree or higher 61%; Employment status: both parents employed 69%	MVPA and average counts/min (accelerometer), duration of PA behaviors in specific contexts and school-related active travel (self-reported)	MVPA and average PA counts/min were associated with walkability in adolescents residing in low SES areas only. Walking for transport during leisure time was negatively associated with neighborhood SES.	3
Dessing, de Vries [49]; The Netherlands	184; 53%; 8–12 y	NR	Built environment characteristics (comparison of variables between route measures) Active travel to school	Children mainly traveled through residential areas on their way to school (>80% of the route). Actual walking routes had less traffic area, greater % water along route, fewer street lights/km, fewer zebra crossings, and lower % sidewalk along the route than estimated routes. Actual cycling routes had greater % recreational area, % water along route, traffic lights/km, junctions/km, % residential streets, fewer trees/km, street lights/km, street bumps/km, and zebra crossings/km, and lower % sidewalk along route and % pedestrian path than estimated routes.	3
DeWeese, Ohri-Vachaspati [50]; USA	404; 48%; 3–18 y	Ethnicity: Non-Hispanic black 49%, Hispanic 44%, Non-Hispanic Caucasian 7%; Highest parental education: some college or higher education 31%; Average block group median income (mean ± SD): ~USD36,900 ± 16,200	Parent reported PA behaviors (categorized as 60-min of PA on 7 days per week vs. <7 days; ever walked or biked to school vs. never; walked to destinations often vs. sometimes, rarely, or never)	Three classes were identified and characterized: (1) “Low PA-Low Food” (N = 72, 17% of sample) had the lowest probability for above-median residential dwellings and intersections, and for the presence of a PA facility, supermarket, small grocery store, convenience store, and fast-food restaurant and a high probability of the presence of a large park; (2) “High Intersection & Parks-Moderate Density & Food” (34%) had the highest probability for above median intersections and for the presence of large parks, and low probabilities of having a PA facility, a supermarket, and a small grocery store; (3) “High Density- Low Parks-High Food” (49%) had the highest probability of above-median residential dwellings and the presence of PA facilities, supermarkets, small grocery stores, convenience stores, and fast-food restaurants, and had the lowest probability for a large park presence. Children in the High Density-Low Parks-High Food class had higher odds of walking or biking to school and to other destinations compared to children in the Low PA-Low Food class, before adjusting for covariates. Neither healthy nor unhealthy food intake differed across classes.	5
Helbich, Emmichoven [51]; The Netherlands	97; 60%; 6–11 y	NR	Active travel to/from school (estimated from GPS), environmental characteristics of school route travelled	Active school travel was negatively associated with distance when only personal, traffic safety, and weather features were considered. After adjusting for urban environments, the distance to school was not significant; well-connected streets and % cycling lanes were positively associated with active school travel.	2
Hinckson, Cerin [52]; Aotearoa New Zealand	524; 55%; 12–18 y	50% resided in high-income neighborhoods; Ethnicity: Māori (indigenous to Aotearoa New Zealand) 3%, New Zealand European 70%, Pacific 2%, Asian 12%, Other 13%; Household highest educational attainment: post-school qualification or higher 70%	MVPA and sedentary time (average min/day, accelerometer)	MVPA was associated with residential density and number of parks within 2 km from home independently, and also when combined into an objective environmental index of activity-friendliness.	3
Ikeda, Hinckson [53]; Aotearoa New Zealand	542; 51%; 8–13 y	Ethnicity: Māori 12.9%, New Zealand European 52.7%, Pacific 15.3%, Asian 15.0%; Education level: bachelor’s degree or higher 30.0%; Car ownership: >1 63.8%	Usual mode of travel to school (self-reported and dichotomized to active or passive travel)	Active school travel was negatively associated with the distance to school. Full mediation of the association between the active mobility environment occurred through the distance to school. All indicators of the active mobility environment were negatively correlated with the distance to school.	4
Islam, Moore [54]; Bangladesh	109; 39%; 9–14 y	Monthly household incomes: Taka25,001–40,000 41% (urban average household monthly income: Taka9878); Education level: bachelor’s degree (father) 59%, bachelor’s degree (mother) 50%; Average residency: 6.5 y	Children’s outdoor activities (self-reported average time outdoors in past week, calculated using reported start and end times of outdoor activities within the neighborhood)	Average time outdoors on weekdays was negatively associated with total building footprint area within the neighborhood.	4
Jauregui, Soltero [55]; Mexico	1191; 53%; 6–14 y	Household income: Mexican peso <5000 50% (income data available for 59% of participants)	Active school travel (parent-reported usual mode of travel to school, walking or biking classified as active school travel)	Active school travel was associated with lower walkability in the 400 m buffer only.	3
Kyttä, Broberg [15]; Finland	1837; 49%; 10–15 y	Housing: detached house 37%, apartment building 33%, terraced house 30%; Household car ownership: 92%	Active school travel (self-reported walking or cycling normally used on journeys both to and from school), parental licenses for their child’s independent mobility, and territorial range (distance to the furthest marked place the child travelled to independent of parental supervision), dichotomized as above versus below the within-age group mean	Active school travel was positively related to residential density, and negatively related to the proportions of green space and child population. The distance from home to children’s meaningful places decreased as the residential density increased and increased as the proportion of green space and child population increased. Meaningful places of children were located close to home: 16.6% were ≥ 50 m from home, 24.8% were within 100 m, and 53.3% within 0.5 km of home. The size of the territorial range was significantly higher in more green areas, and lower in areas with a higher child population. Children had significantly more limitations on mobility licenses if the child’s home was in a more densely built area. A significant correlation was found between the number of marked destinations and school travel mode. The size of territorial range was positively correlated with active school travel mode.	3
Laxer and Janssen [56]; Canada	6626; 50%; 11–15 y	Household SES: high 24%, medium-high 32%, low-medium 35%, low 10%; Ethnicity: Caucasian 73%, Other 27%	PA (self-reported usual and past 7 days number of days physically active for ≥60 min/day, dichotomized as physically inactive (≤4 days/week) or physically active (>4 days/week))	Physical inactivity was higher in neighborhoods with higher walkability, lower cul-de-sac density, and moderate to high park space. An estimated 23% of physical inactivity within the population was attributable to living in walkable neighborhoods, 16% was attributable to living in neighborhoods with a low density of cul-de-sacs, and 15% was attributable to living in neighborhoods with a moderate to high amount of park space.	3
McGrath, Hinckson [57]; Aotearoa New Zealand	226; 51%; 5–13 y	Income per adult (mean ± SD): NZD39,000 ± 2000; Ethnicity: Māori/Polynesian 17%, European/other 72%, Asian 11%	School travel mode (parent-reported 7-day travel log), classified as passive (car or bus) or active (walk, bicycle, skateboard, or scooter); MVPA, step-based MVPA, hourly step counts (accelerometer)	MVPA steps on non-school days were associated with living in neighborhoods with more green space (positive) and food outlet density (negative).	5
Mecredy, Pickett [58]; Canada	8535; NR; 11–15 y	NR	MVPA outside of school (self-reported usual hours of exercise in free time) categorized as ≥4 h/week or <4 h/week.	Higher MVPA was associated with residing in neigborhoods with the highest street connectivity quartile.	5
Mitchell, Clark [59]; Canada	435; 59%; 9–14 y	Median family income: CAD71,758	Average daily MVPA during non-school hours (accelerometer)	MVPA out of school hours was associated with parks with sports fields and multi-use path space at both buffers in grouped analyses. Significant associations were observed between boys’ MVPA and parks with sports fields (positive) and parks with playgrounds (negative) at both buffers (although the magnitudes were greater for 800 m), and girls’ MVPA and parks with sports fields (positive, 800 m buffer only).	3
Mölenberg, Noordzij [60]; The Netherlands	1841 (N = 1607 for outdoor play, N = 1545 for sedentary behavior); 56% (intervention group), 49% (control group); Mean 6 y at wave 1, 9.7 y at wave 2	Net household income/month: ≤€2000 18% (intervention) and 15% (control), >€2000–€3200 34% (intervention) and 27% (control), >€3200 48% (intervention) and 58% (control); Ethnicity: Dutch 60% (intervention) and 70% (control), Other Western 13% (intervention) and 12% (control), Non-Western 27% (intervention) and 18% (control); Maternal education: mid-high 54% (intervention) and 63% (control); Paternal education: mid-high 54% (intervention) and 62% (control)	Outdoor play (parent-reported exercise at school and outside school hours for an average week, calculated as mean min/week playing outdoors), sedentary behavior (parent-reported television viewing and computer game use for an average week, calculated as mean min/week watching television and computer gaming	The introduction of a dedicated PA space within 600 m from home, and the reduction in the distance per 100 m, did not affect outdoor play or sedentary behaviors.	4
Nordbø, Raanaas [61]; Norway	21,146; 49%; 8 y	Maternal university education: 78%	Leisure-time PA (parent-reported time in PA outside school hours), classified as ≥5 h/week or ≤ 4 h/week. Organized PA participation (parent-reported days/week participation in any kind of organized leisure PA), classified as ≥2 days/week or once a week or less. Informal social activity with friends and peers (parent-reported time with friends and peers, excluding school hours and organized activities), classified as ≥2 days/week or once a week or less.	Leisure-time PA was associated with having a park within 800 m from home (summer) and living in a neighborhood with a higher proportion of green space (winter). Participation in organized and social activities was associated with population density and access to facilities.	4
Oliver, Badland [62]; Aotearoa New Zealand	217; 49%; 6–15 y	Average annual household income: <NZD60,000 39%, NZD60,001–100,000 25%, >NZD100,000 25%; Ethnicity: Māori 24%, Asian 15%, New Zealand European/Other 60%; Unlimited car access: 87%; Residing in school zone: 56%; Parent neighborhood self-selection: prefer high walkable and live low walkable 31%, prefer high walkable and live high walkable 20%, prefer low walkable and live low walkable 31%, prefer low walkable and live high walkable 19%	Active school travel (self-reported walking or cycling normally used on journeys both to and from school), parental licenses for their child’s independent mobility, and territorial range (distance to the furthest marked place the child travelled to independently)	Active school travel was significantly associated with the city a child lived in and neighborhood self-selection (children who lived in a low-walkable neighborhood, but whose parents preferred a highly walkable neighborhood were three times less likely to have active school travel than their counterparts), and negatively associated with distance to school.	3
Sallis, Cain [63]; USA	3677 (758 children aged 6–11 y and 897 adolescents aged 12–16 y (findings for these age groups presented here)); NR; 6 y and older	Based on recruitment strategy, approximately half of the participants should be from low income neighborhoods, and half from high income neighborhoods; Ethnicity: Non-Caucasian 31% (children) and 33% (adolescents)	MVPA (accelerometer; mean daily hours out-of-school hours for adolescents, mean daily MVPA in neighborhood for children (via temporal matching of accelerometer and parent reported times in neighborhood locations)). Active travel to common locations (parent-reported for children, self-reported for adolescents).	Controlling for GIS-derived macro-level walkability, total microscale environment scores were significantly related to active travel in both groups, and with leisure-time PA and accelerometer measures in children.	3
Sallis, Conway [64]; USA	928; 50%; 12–16 y	Based on the recruitment strategy, approximately half of the participants should be from low income neighborhoods, and half from high income neighborhoods; Ethnicity: Non-Hispanic Caucasian 66%; Parent education: college degree or higher 74%; Time living at current address (mean ± SD): 12.6 ± 7.0 y; Motor vehicles/licensed driver (mean ± SD): 1.1 ± 0.38	MVPA, sedentary time (accelerometer), self-reported active travel to school and non-school destinations (e.g., recreation facility, friend’s house, park, food outlet), leisure-time PA in specified locations, number of days accumulated 60 min PA, number of sports and PA classes outside of school, usual time/day in sedentary behaviors	Walkability was positively related to objectively measured PA and walking for transportation. Self-reported sedentary time and television time were negatively related to walkability. The time in vehicles was negatively related to walkability only among those living in higher income census blocks.	5
Tucker, Irwin [65]; Canada	811; 50%; 11–13 y	Household income: <CAD50,000 21%, CAD50,000–69,999 13%, >CAD70,000 32%; Ethnicity: Caucasian 75%, Black 2%, Latin-American 7%, Asian 6%, Other 9%; Paternal education: college or higher 70%; Maternal education: college or higher 70%	PA (self-reported type and intensity of activity in 30 min blocks throughout the afternoon and evening of the previous day (15:00–23:00) plus blocks of time for morning and afternoon recess, lunch time, and physical education class (with 15 min blocks allocated for the morning and afternoon recess and 30 min blocks allotted for lunch hour and physical education class)).	MVPA was associated with having ≥2 recreational opportunities in the neighborhood	4
van Loon, Frank [66]; Canada	366; 53%; 8–11 y	Ethnicity: European/North American 44%, East/Southeast Asian 29%, South Asian 11%, Mixed/other 16%	MVPA (daily average, accelerometer)	MVPA was positively associated with commercial density, residential density, number of parks, and intersection density; and negatively associated with the distance to school and recreation sites. When entered as a composite index, these measures accounted for 4.4% in the variation in MVPA for the full sample. Sex-stratified models better explained the relationships between the neighborhood environment and PA. For boys, built and social environment characteristics of neighborhoods accounted for 8.7% of the variation in MVPA, and for girls, neighborhood factors explained 7.2% of the variation. Sex stratified models also point towards distinct differences in factors associated with PA, with MVPA of boys associated with wider-ranging neighborhood characteristics than MVPA of girls. For girls, two safety-related neighborhood features were found to be significantly associated with MVPA: cul-de-sac density and proportion of low speed limit streets.	4
Villanueva, Giles-Corti [67]; Australia	926; 50%; 10–12 y	School-level SES: low 28%, medium 34%, high 38%; Maternal education: less than secondary education 28%, secondary education/trade/diploma 56%, Bachelor degree or higher 16%	Activity spaces: associations included average daily steps (pedometer) and leisure-time PA (parent-reported time in leisure-time PA in previous week)	Activity space size was positively associated with the confidence to travel independently and negatively associated with utilitarian destination availability. For boys, activity spaces were larger if they owned a bike. For girls, activity space size was positively associated with being independently mobile, leisure time PA, and parent confidence in their ability to travel independently, and negatively associated with parents reporting living on a busy road.	3
Wang, Conway [68]; USA	928; 50%; 12–16 y	Based on the recruitment strategy, approximately half of the participants should be from low income neighborhoods, and half from high income neighborhoods; Ethnicity: Non-Hispanic Caucasian 66%; Parent education: college degree or higher 74%; Time living at current address (mean ± SD): 12.6 ± 7.0 y; Motor vehicles/licensed driver (mean ± SD): 1.1 ± 0.38	Active travel to/from school and non-school destinations (e.g., recreation facility, friend’s house, park, food outlet), active transport index (sum of z scores for active travel to school and non-school destinations)	GIS-derived neighborhood walkability and the count of nearby parks and recreation facilities (as well as audited streetscape quality using MAPS) had significant main effects in the direction of more PA-supportive built environments associated with more active travel. Significant two-way interactions with GIS-based measures were observed: self-efficacy × GIS- based walkability index, and self-efficacy × GIS-based counts of parks and recreation facilities. In each two-way interaction, the highest active travel was found among adolescents, with the combination of the PA-supportive built environment and positive psychosocial characteristics.	4

* Relevant to GIS analyses/measures. Add Health = National Longitudinal Study of Adolescent Health, AS!BC = Action Schools! British Columbia, BMI = body mass index, GED = graduate equivalency degree, GIS = geographic information system, GPS = global positioning system, HBSC = Health Behavior in School-aged Children Survey, IDEFICS = Identification and prevention of Dietary- and lifestyle-induced health EFfects in Children and infantS, MAPS = Microscale Audit of Pedestrian Streetscapes, MVPA = moderate-to-vigorous intensity physical activity, NfAK = Neighborhoods for Active Kids, NIK = Neighborhood Impact on Kids, NR = not reported, PA = physical activity, PACE = Patient-Centered Assessment and Counseling for Exercise, SD = standard deviation, SES = socio-economic status, SPACES = Studying Physical Activity in Children’s Environments across Scotland, STEAM = Spatial Temporal Environment and Activity Monitoring, TEAN = Teen Environment and Neighborhood, TREK = Travel Environment and Kids, URBAN = Understanding the Relationship between Activity and Neighborhoods, y = years.
